# Sequential Meals Containing Animal and Plant-Based Saturated Fats Have Differential Effects on Postprandial Gut Hormones but No Impact on Satiety Compared with Unsaturated Fats in Generally Healthy Males: Findings from the Randomized Controlled Crossover CocoHeart Study^☆^

**DOI:** 10.1016/j.tjnut.2025.06.027

**Published:** 2025-07-01

**Authors:** Gloria Wong, Miriam E Clegg, Damian Ross, Julie A Lovegrove, Kim G Jackson

**Affiliations:** Hugh Sinclair Unit of Human Nutrition, Institute for Cardiovascular and Metabolic Research and Institute for Food Nutrition and Health, University of Reading, Reading, United Kingdom

**Keywords:** appetite, butter, coconut oil, gastrointestinal hormones, postprandial lipemia

## Abstract

**Background:**

Saturated fat (SFA)-rich meals are often linked to elevated postprandial triacylglycerol responses compared with unsaturated fats. Despite the growing popularity of coconut oil in the United Kingdom diet, effects of this SFA-rich oil on postprandial lipemia and physiological appetite responses are unclear.

**Objectives:**

This study compared sequential high-fat test meals rich in butter and coconut oil with a vegetable oil blend (safflower and olive oil) on postprandial triacylglycerol (primary outcome), lipids, glucose, and gut hormones responses, and physiological measures of appetite in healthy males.

**Methods:**

In a single-blind, randomized acute 3-armed crossover study, 13 males (53 ± 3 years, body mass index 24.4 ± 3.0 kg/m^2^) consumed sequential test meals containing SFA-rich oils/fats or a vegetable oil blend (breakfast 53.6 g and lunch 33.6 g fat) on 3 occasions, each separated by a 4-wk period. Blood samples and satiety ratings were collected prior to and at regular intervals over 480 min post-test breakfast. Blood pressure and arterial stiffness were measured at 0, 150, 300, and 480 min. Postprandial data were analyzed using linear mixed models and satiety ratings using analysis of covariance.

**Results:**

Postprandial triacylglycerol, glucose, insulin, ghrelin, blood pressure, and arterial stiffness or perceived satiety responses were similar between the test fat/oils. The incremental area under the curve (iAUC) for the postprandial glucose-dependent insulinotropic polypeptide response was higher with vegetable oil compared with the SFA-rich meals whereas the glucagon-like peptide-1 response was lower after the butter than coconut and vegetable oil-rich meals (*P* ≤ 0.012). The iAUC for the peptide YY response was lower after butter than coconut oil-rich meals (*P* ≤ 0.048), but not different compared with vegetable oil.

**Conclusions:**

Despite varying fatty acid compositions, postprandial triacylglycerol responses were similar between fats/oils. Our findings suggest that butter and coconut oil have differential effects on gut hormone responses compared with unsaturated fats without an impact on satiety in generally healthy males.

**Trial registration number:**

NCT05264233.

## Introduction

Elevated postprandial triacylglycerol (TAG) concentrations are an independent modifiable cardiovascular disease (CVD) risk factor [1], likely mediated by adverse effects on several mechanisms including circulating lipid and remnant lipoprotein metabolism [2] and vascular function [3,4]. The characteristics of dietary fat, particularly the degree of saturation and chain length, can affect both the magnitude and duration of the postprandial TAG response. Studies have found that meals rich in unsaturated fatty acids often favor the reduction of postprandial lipemia compared to SFA [[5], [6], [7], [8], [9]]. However, this is not always the case [10], suggesting that the source of SFA in a meal might be an important factor in relation to CVD risk. Coconut oil, a plant-derived SFA, is becoming increasingly popular in Western diets. This oil contains ∼90% SFA, of which over half are medium-chain fatty acids (C8-12, MCFA) and comprise predominately of lauric acid (C12:0). Although chemically defined as a MCFA, lauric acid in coconut oil is endogenously metabolized as a long-chain fatty acid with only 20%–30% transported to the liver as MCFA, whereas the remaining fatty acids are packed into chylomicrons [11]. Studies have shown that 30–40 g of coconut oil provokes a lower postprandial TAG response when compared with animal-derived SFA (butter and lard) or other plant-derived SFA higher in long-chain fatty acids (palm oil [12] and palm oil-rich blends [13]) in healthy subjects whereas a greater response has been observed after consuming 20 g of coconut oil relative to corn oil in participants with obesity [14]. Others have demonstrated no difference in postprandial lipemia between coconut oil and animal-derived SFA (tallow and milk fat) with n-6 PUFA or MUFA-rich oils [[15], [16], [17]].

Meal fatty acid composition has also been shown to impact the satiating properties of foods. Kozimor et al. [18] demonstrated that SFAs (mixture of butter, red palm oil, and coconut oil) resulted in greater fullness and lower prospective food consumption compared with MUFA (mixture of canola oil and extra virgin olive oil) and PUFA (mixture of sunflower oil and flaxseed oil) and Yao et al. [19] showed that coconut oil resulted in greater fullness ratings from the visual analog scale (VAS) than olive oil over a 300 min period. Both fatty acid chain length and saturation play a role in the release of gut hormones involved in appetite control. A comprehensive review by Kaviani and Cooper [20] reported that meals rich in n-6 PUFA induced greater gut hormone [glucagon-like peptide-1 (GLP-1), peptide YY (PYY), and glucose-dependent insulinotropic polypeptide (GIP)] responses, followed by MUFA and then SFA. Compared with coconut oil-rich meals, corn oil has been shown to increase postprandial PYY concentrations in adolescents with obesity [14] whereas a trend toward an increase in plasma PYY was observed compared with olive oil in normal weight adults. In the latter study, the coconut oil-rich breakfast was also associated with reduced hunger and desire to eat [21]. However, studies comparing coconut oil with other dietary fats varying in fatty acid chain length and saturation on gut hormones and satiety are limited. To address this knowledge gap, the aim of this study was to determine the acute effects of sequential meals containing 80 g of fat from different dietary SFA sources varying in composition (butter and coconut oil) with a vegetable oil blend (*n*-9 MUFA/*n*-6 PUFA source) on postprandial lipids, glucose, gut hormones, and self-reported appetite responses. It was hypothesized that meals rich in coconut oil and butter would show similar postprandial TAG (primary outcome) and physiological appetite responses compared with the vegetable oil blend.

## Methods

### Subjects and study design

Caucasian males aged between 30 and 70 y and BMI (in kg/m^2^) of 19–32 were recruited, and the single-center study was conducted at the Hugh Sinclair Unit of Human Nutrition, University of Reading between December 2019 and April 2021 (this study was suspended for 9 mo in 2020 due to the COVID-19 pandemic). All subjects met the inclusion criteria with a fasting total serum cholesterol <7.5 mmol/L, TAG <2.3 mmol/L, and glucose <7.0 mmol/L. Subjects were excluded if they were smokers, had a history of myocardial infarction or stroke in the past 12 mo; kidney, liver, pancreas or gastrointestinal disorder; hypertension (blood pressure >140/90 mm Hg), cancer, taking medication for hyperlipidemia (e.g., statins), hypertension, inflammation or prescribed antibiotics within the last 3 mo; drinking in excess of 14 units of alcohol per week, anemic (<130 g/L hemoglobin), or planning a weight-reducing regime or taking any dietary supplements known to influence lipids (e.g., plant stanols and fish oil) or any other unusual medical history or diet and lifestyle habits. Our participants were considered generally healthy as they self-reported being disease-free (i.e., not diagnosed with any chronic conditions), and their screening results were within clinical reference ranges. The University of Reading Research Ethics Committee gave a favorable ethical opinion for the conduct of the human study (UREC reference number: 19/30), and it was performed in accordance with the Declaration of Helsinki guidelines. In line with our previous studies, a sequential test meal approach was used to mimic a more representative pattern of food intake in a Western dietary pattern. Since sex has been reported to contribute to the interindividual variability in the postprandial TAG response to a high-fat meal as well as gut hormone responses [22], we chose to include only males in the current study and use a crossover design so that each participant could act as their own control. This approach has been used in our previous postprandial studies [9,23,24].

A single-blind, randomized acute 3-armed crossover study was carried out in which healthy males were assigned to sequential high-fat meals rich in butter, coconut, or a mixture of vegetable oils in random order on 3 occasions, each separated by 4 wk. This washout period was in line with our previous postprandial studies in which test fats rich in SFA, *n*-6 PUFA, and MUFA were incorporated [9,23]. The participants were randomly assigned to a sequence to receive the test fat/oils during the 3 study visits. Randomization without stratification was conducted by a single study researcher (GW) using the Research Randomizer tool (https://www.randomizer.org). Only participants were blinded to the specific fat/oil consumed at each visit because this was incorporated into a warm chocolate drink so that the test breakfast and lunch meals appeared similar. Prior to the first study visit, a 4-d weighted food diary was completed to assess habitual dietary intake. Subjects were also required to abstain from vigorous exercise and alcohol consumption 24 h prior to the study day and to consume a low-fat evening meal (<10 g total fat) provided by the researchers. From 20:00 onward, subjects fasted overnight, drinking only water during that time until they arrived at the Hugh Sinclair Unit of Human Nutrition at ∼08:00. Body composition was measured, and a finger-prick blood sample was collected to ensure that hemoglobin was ≥130 g/L prior to cannula insertion into the antecubital vein of the forearm. Two fasting blood samples (–30, 0 min) were collected before the first test meal was provided and then sequentially at 30, 60, 90, 120, 180, 240, 300, 330, 360, 390, 420, and 480 min. Subjects were given a test breakfast (toasted white bread with jam and a warm chocolate drink containing 50 g of fat from the test fat/oils, 53.6 g total fat content) at 0 min and lunch (toasted white bread with jam and a warm chocolate drink containing 30 g of fat from the test fat/oil, 33.6 g total fat content) at 330 min. Table 1 [25] presents the fatty acid composition of the test fat/oils, and Table 2 presents the nutritional composition of the test meals. The test fat/oils used in the study included butter (farmhouse salted butter, Wyke), coconut oil (organic refined odorless coconut oil, TIVI, Friends of Health) and a 50:50 mixture of safflower oil (organic refined safflower oil, Spectrum Culinary) and olive oil (refined virgin olive oil, Filippo Berio).

**TABLE 1:** Fatty acid composition of the coconut oil, butter, and vegetable oils (g/80 g fat).

	Coconut oil	Butter	Vegetable oil^1^
C4:0	—	3.38	—
C6:0	0.32	1.98	—
C8:0	5.84	1.11	—
C10:0	5.28	2.39	—
C12:0	38.24	2.74	—
C14:0	14.48	8.52	—
C15:0	—	0.86	—
C16:0	7.12	22.46	—
C17:0	—	0.57	—
C18:0	2.16	8.72	—
C20:0	0.08	0.14	—
C22:0	—	0.06	—
C24:0	—	0.01	—
cis C10:1	—	0.24	—
cis C12:1	—	0.06	—
cis C14:1	—	0.70	—
cis C15:1	—	0.01	—
cis C16:1	—	1.26	0.29
cis C17:1	—	0.22	0.04
cis C18:1	5.12	16.09	30.11
cis C20:1	—	0.17	0.12
C22:1	—	—	0.08
C24:1	—	—	0.04
cis C24:1	—	0.08	—
C18:2	—	—	34.10
cis n-3 C18:3	—	0.46	0.29
cis n-6 C18:3	—	0.02	—
cis n-3 C18:4	—	0.02	—
cis n-6 C20:3	—	0.06	—
cis n-6 C20:4	—	0.08	—
cis n-3 C20:5	—	0.06	—
cis n-3 C22:5	—	0.08	—

1 Vegetable oil contains a 50:50 mixture of safflower oil and olive oil.

**TABLE 2:** Nutritional composition of the sequential high-fat test meals incorporating the different test fat/oils^1^.

	Carbohydrate (g)	Fat (g)	Protein (g)	Energy (kJ)
Breakfast				
Test fat/oils	0.0	50.0	0.0	1848
Skimmed milk (150 g)	8.0	0.5	5.0	220
Nesquik (15 g)	12.0	0.5	0.6	240
Skimmed milk powder (15 g)	8.0	0.5	5.0	220
White bread (105 g)	49.0	2.1	8.5	1002
Jam (30 g)	21.0	0.0	0.1	335
Total	98.0	53.6	19.2	3865
Lunch				
Test fat/oils	0.0	30.0	0.0	1109
Skimmed milk (150 g)	8.0	0.5	5.0	220
Nesquik (15 g)	12.0	0.5	0.6	240
Skimmed milk powder (15 g)	8.0	0.5	5.0	220
White bread (105 g)	49.0	2.1	8.5	1002
Jam (30 g)	21.0	0.0	0.1	335
Total	98.0	33.6	19.2	3126

1 Determined from manufacturers’ data.

#### Assessment of body composition and blood pressure measurements

On each postprandial test day, body composition was measured using the Tanita BC-418 digital scale (Tanita Europe), under standard body type setting, with 1 kg removed for clothing. Blood pressure (systolic blood pressure, diastolic blood pressure, pulse pressure, heart rate and mean arterial pressure) and arterial stiffness (augmentation index and pulse wave velocity) were determined on the right arm in the supine position using the IEM Mobil-O-Graph (Numed Healthcare) device at baseline (0 min), 150, 300, and 480 min.

### Biochemical analysis

Blood samples were collected into serum separator clot activator tubes (VACUETTE; Greiner Bio-One) and allowed to sit at room temperature for 30 min prior to centrifugation at 1750 × *g* for 15 min at 4°C. After centrifugation, serum samples were aliquoted and then stored at –20°C for subsequent analysis. Serum TAG, non-esterified fatty acids (NEFA), glucose, total cholesterol (fasting only), HDL-cholesterol (fasting only) and C-reactive protein (fasting only) concentrations were measured in all of the blood samples [fasting (0 min), 30, 60, 90, 120, 180, 240, 300, 330, 360, 390, 420, and 480 min] using a RX Daytona Plus clinical chemistry analyzer (Randox Laboratories). The fasting LDL-cholesterol concentration was calculated using the Friedewald formula [26].

Blood samples for the gut hormone measurements were collected into tripotassium EDTA tubes (VACUETTE; Greiner Bio-One) and pretreated with 50 mmol of dipeptidyl peptidase-IV prior to centrifugation at 1750 × *g* for 15 min at 4°C. After centrifugation, plasma samples were then aliquoted and stored at –80°C until analysis. The MILLIPLEX MAP Human Metabolic Hormone Magnetic Bead Panel (EMD Millipore Corporation) was used for measurement of ghrelin, GIP, GLP-1, PYY, and insulin at selected time points (0, 30, 60, 120, 240, 330, 360, 390, and 480 min) by the Luminex 200 system with xPONENT software 3.1 (Diasorin).

### Satiety measurement

Perceived satiety was assessed using a 100 mm VAS to measure hunger, satisfaction, fullness, desire to eat, and prospective consumption at baseline (0 min) and every 30 min after breakfast throughout the 480 min period. The VAS was marked as “not at all” (0) and “extremely” (100). The VAS ratings were quantified by measuring the distance (in mm), between the left end of the scale (0 mm) and the point marked by the participant.

### Power calculation and statistical analysis

Due to the limited number of studies incorporating meals rich in coconut oil, data from the systematic review and meta-analysis of meal fatty acids on postprandial lipemia were used to calculate the sample size for this study [27]. The mean expected change in the incremental TAG response between SFA and unsaturated fatty acids was 67.7 mmol/L × min. With 90% power, 5% significance level, and SD of 47.0, the minimum number of participants required in this crossover study was 13. To allow for a 15% dropout rate, a sample size of 15 participants was needed. A *P* value of ≤0.05 was considered significant for the primary outcome measure (postprandial TAG response) and secondary outcomes which included postprandial NEFA, glucose, gut hormones (ghrelin, GIP, GLP-1, PYY, and insulin), blood pressure (systolic blood pressure, diastolic blood pressure, pulse pressure, heart rate and mean arterial pressure), measures of arterial stiffness (augmentation index and pulse wave velocity), and perceived measures of satiety (hunger, satisfaction, fullness, desire to eat and prospective consumption).

All statistical analyses were conducted using SPSS version 29 (IBM Corp.). Normality of the data was assessed using the Shapiro–Wilk test. Variables with non-normal distributions (TAG, NEFA, glucose, gut hormones, perceived satiety ratings, and blood pressure) were log-transformed prior to analysis. Model assumptions were assessed via visual inspection of residual and Q–Q plots. Histograms and boxplots were also used to screen for skewness and outliers. For the analysis of the VAS satiety ratings using analysis of covariance (ANCOVA), the assumptions of linearity and homogeneity of regression slopes were also verified.

Postprandial responses were summarized using AUC, calculated via the trapezoidal method, and incremental AUC (iAUC), defined as iAUC = AUC – (mean fasting concentration × duration). Given the initial suppression in NEFA following the test meal, AUC and iAUC for NEFA were calculated from 90 to 480 min, corresponding to the timing of the average postprandial minimum concentration and end of sampling period. For all primary and secondary outcomes apart from the VAS satiety ratings, linear mixed model analyses were implemented to examine the effects of the different test fat/oil on fasting and postprandial summary data (AUC and iAUC). Postprandial time course profiles to the different test fat/oils were also analyzed using linear mixed models. Fixed effects included test fat/oils, time, period, and the test fat/oils × time interaction, with participant ID included as a random effect regardless of their degrees of significance. The VAS parameters were analyzed using 1-way ANCOVA at each postprandial time point, with test fat/oils as the fixed factor and the corresponding baseline (0 min) value as the covariate, as recommended by Blundell et al. [28]. Bonferroni correction was applied for multiple comparisons in posthoc testing. Data are presented in the tables and figures as unadjusted and untransformed means ± SEM, unless otherwise stated.

## Results

### Subject characteristics

**FIGURE 1:**
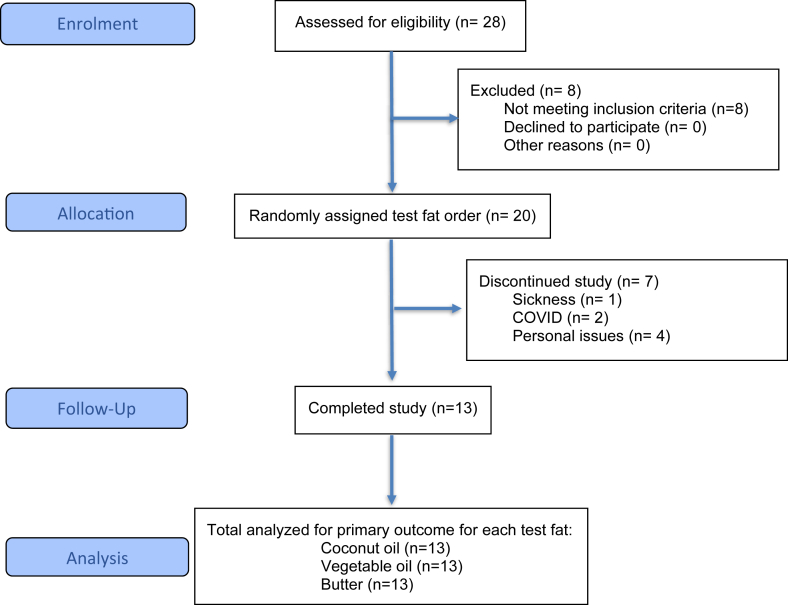
Flow of participants through the CocoHeart study.

Among 24 screening visits conducted at the University of Reading, 20 subjects were found to be eligible, and 7 subjects withdrew due to various reasons, including sickness (*n* = 1), COVID-related issues (*n* = 2), and for personal reasons (*n* = 4). Due to the COVID-19 pandemic, a higher dropout rate than expected was observed (35% compared with 15%). A total of 13 Caucasian males, with a mean age of 53 ± 3 y and a BMI of 24.4 ± 0.8 successfully completed the 3 postprandial study visits (Figure 1), and data were available for *n* = 13 for all outcomes apart from PYY (*n* = 12). The baseline (fasting) BMI, blood pressure, arterial stiffness, biochemical data, and gut hormones measured on each study visit are shown in Table 3.

**TABLE 3:** Participant baseline measures at the beginning of each acute study visit^1^.

Characteristics	Butter	Vegetable oil	Coconut oil	*P* value^2^
BMI (kg/m^2^)	24.4 ± 2.8	24.5 ± 2.6	24.3 ± 2.6	0.991
Blood pressure				
Systolic (mm Hg)	121 ± 2	120 ± 3	123 ± 3	0.501
Diastolic (mm Hg)	84 ± 2	81 ± 3	84 ± 2	0.469
Pulse pressure (mm Hg)	37 ± 2	37 ± 2	39 ± 2	0.759
MAP (mm Hg)	101 ± 2	99 ± 3	103 ± 2	0.452
Arterial stiffness				
Reflection magnitude (%)	64.4 ± 1.7	63.3 ± 2.9	67.5 ± 1.8	0.718
Augmentation index (%)	15.5 ± 4.9	9.5 ± 3.3	11.8 ± 3.0	0.458
PWV (m/s)	7.61 ± 0.36	7.68 ± 0.36	7.85 ± 0.39	0.860
Fasting biochemical profile				
Total cholesterol (mmol/L)	4.88 ± 0.29	4.74 ± 0.25	4.93 ± 0.22	0.919
LDL-cholesterol (mmol/L)	3.27 ± 0.25	3.16 ± 0.19	3.32 ± 0.18	0.927
HDL-cholesterol (mmol/L)	1.39 ± 0.08	1.37 ± 0.10	1.39 ± 0.10	0.975
Triacylglycerol (mmol/L)^3^	1.12 ± 0.15	1.10 ± 0.16	1.10 ± 0.11	0.994
C-reactive protein (mg/L)	1.33 ± 0.35	2.21 ± 1.13	1.13 ± 0.29	0.545
Glucose (mmol/L)^3^	4.96 ± 0.10	4.97 ± 0.11	5.20 ± 0.09	0.954
NEFA (μmol/L)^3^	412 ± 31	540 ± 77	468 ± 54	0.407
Gut hormones				
Ghrelin (ng/L)	93.0 ± 15.0	87.1 ± 14.1	96.0 ± 22.9	0.740
GIP (ng/L)	31.8 ± 3.0	34.2 ± 3.4	35.0 ± 2.5	0.913
GLP-1 (ng/L)	140 ± 9.0	137 ± 9	129 ± 9	0.776
PYY (ng/L)^3^	98.1 ± 29.8	98.1 ± 29.1	95.8 ± 30.1	0.799
Insulin (ng/L)^3^	266 ± 88	253 ± 87	258 ± 88	0.968

Abbreviations: GIP, glucose-dependent insulinotropic polypeptide; GLP-1, glucagon-like peptide-1; MAP, mean arterial pressure; NEFA, nonesterified fatty acids; PWV, pulse wave velocity; PYY, peptide YY.

1 Values are untransformed and unadjusted means ± SEMs, *n* = 13 for all outcomes apart from PYY (*n* = 12).

2 Data were analyzed using linear mixed models to calculate overall treatment effect in postprandial summary measures, with adjustments made for fixed effects of test fat/oils and period. The participant was included as a random effect. *P* ≤ 0.05 was considered a threshold for statistical significance and adjusted for multiple comparisons using the Bonferroni correction.

3 Indicates data were transformed prior to analysis.

### Postprandial blood lipids and glucose

**Figure 2:**
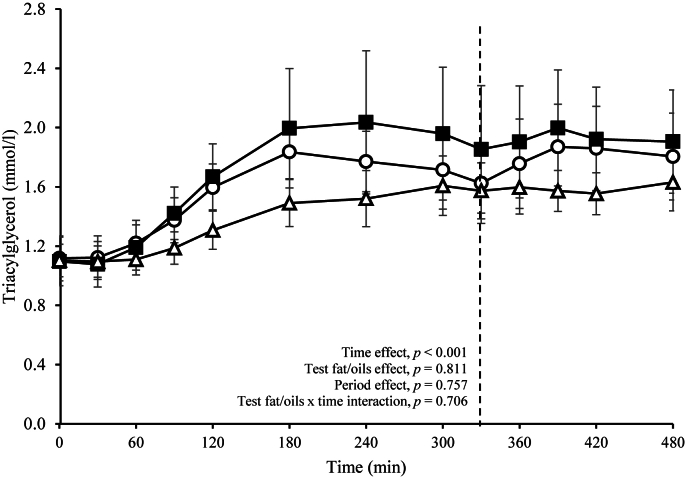
Postprandial serum triacylglycerol (TAG) responses after consumption of sequential test breakfast (0 min) and lunch (330 min) meals containing butter (open circles), vegetable oils (closed squares) and coconut oil (open triangles) in healthy males. Values are untransformed and unadjusted means ± SEMs for *n* = 13 participants. The dotted line (---) represents the timing of the second meal (330 min). A linear mixed model was implemented to examine the effects of the test fat/oils with varying fat composition and time on serum TAG, with adjustment made for the fixed effects of period, time, and test fat/oils. Participant was included as a random effect. *P* ≤ 0.05 was considered a threshold for statistical significance.

There were no significant differences in the time course profiles or the summary measures for the postprandial TAG response (primary outcome) (Figure 2 and Table 4) between the sequential test meals rich in butter, coconut oil, and vegetable oils. NEFA and glucose responses and postprandial summary measures were also similar following the different test fat/oils (Table 4).

**TABLE 4:** Postprandial summary measures for the lipid, glucose, and gut hormones responses in healthy males after sequential test meals rich in butter, vegetable oils, and coconut oil in healthy males^1^.

	Butter	Vegetable oils	Coconut oil	*P* value^2^
TAG (mmol/L × 480 min)				
AUC^3^	789 ± 99	848 ± 154	691 ± 71	0.718
iAUC^3^	253 ± 38	322 ± 96	162 ± 28	0.719
NEFA (mmol/L × 480 min)				
AUC	120 ± 9	130 ± 12	132 ± 7	0.345
iAUC^3^	46.6 ± 16.9	54.6 ± 11.9	48.8 ± 14.0	0.929
Glucose (mmol/L × 480 min)				
AUC	2519 ± 83	2580 ± 71	2663 ± 60	0.890
iAUC	140 ± 72	194 ± 79	166 ± 57	0.871
Ghrelin (μg/L × 480 min)				
AUC^3^	29.4 ± 4.5	24.5 ± 4.7	25.4 ± 4.8	0.755
iAUC^3^	–15.3 ± 3.6	–17.3 ± 3.5	–20.7 ± 6.6	0.064
GIP (μg/L × 480 min)				
AUC	148 ± 10^b^	203 ± 11^a^	126 ± 12^b^	<0.001
iAUC	133 ± 11^b^	187 ± 10^a^	109 ± 12^b^	<0.001
GLP-1 (μg/L × 480 min)				
AUC	114 ± 8	140 ± 10	138 ± 9	0.109
iAUC	47.2 ± 6.5^b^	74.2 ± 7.3^a^	75.7 ± 7.1^a^	0.012
PYY (μg/L × 480 min)				
AUC^3^	62.8 ± 13.0	73.4 ± 12.1	79.9 ± 11.8	0.275
iAUC	15.7 ± 2.8^b^	26.3 ± 3.6^ab^	34.0 ± 6.0^a^	0.029
Insulin (μg/L × 480 min)				
AUC^3^	976 ± 226	937 ± 228	980 ± 224	0.980
iAUC	236 ± 42	234 ± 34	263 ± 42	0.968

Abbreviations: GIP, glucose-dependent insulinotropic polypeptide; GLP-1; glucagon-like peptide-1; iAUC, incremental AUC; NEFA, nonesterified fatty acids; PYY, peptide YY; TAG, triacylglycerol.

1 Values are untransformed and unadjusted means ± SEMs, *n* = 13 for all outcomes except PYY (*n* = 12).

2 Data were analyzed using linear mixed models to calculate overall treatment effect in postprandial summary measures, with adjustments made for fixed effects of test fat/oils and period. Participant was included as a random effect. *P* ≤ 0.05 was considered a threshold for statistical significance and adjusted for multiple comparisons using the Bonferroni correction. Means with different superscript letters within the same row are significantly different between test oil/fats.

3 Indicates data were transformed prior to analysis.

### Postprandial gut hormones and satiety responses

**FIGURE 3:**
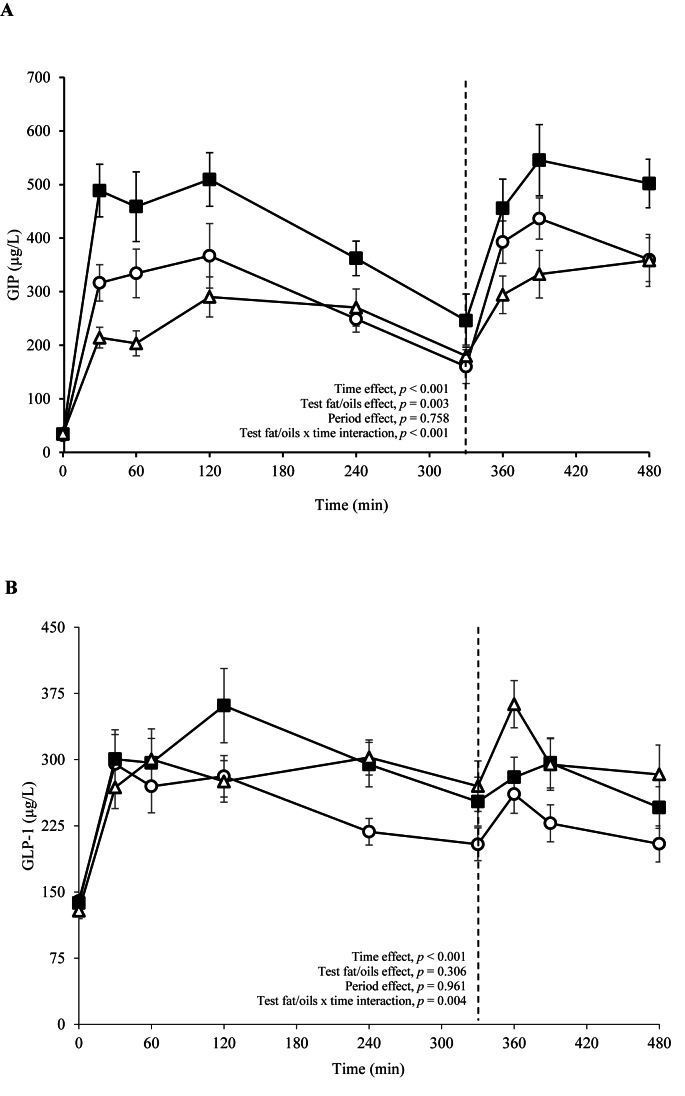
Postprandial plasma (A) glucose-dependent insulinotrophic polypeptide (GIP) and (B) glucagon-like peptide-1 (GLP-1) responses after consumption of sequential test breakfast (0 min) and lunch (330 min) meals containing butter (open circles), vegetable (closed squares) and coconut oil (open triangles) in healthy males. Values are means ± SEMs for *n* = 13 participants and the dotted line (---) represents the timing of the second meal (330 min). Linear mixed model analysis was implemented to examine the effects of test fat/oils with varying fat composition and time on plasma GIP and GLP-1 responses, with adjustment made in the cases for fixed effects of period, test fat/oils and time. Participant was included as a random effect. *P* ≤ 0.05 was considered a threshold for statistical significance.

A significant test fat/oils by time interaction was evident for the postprandial GIP time course profile (*P* < 0.001; Figure 3A), with the AUC and iAUC found to be higher after the vegetable oil-rich meals than both coconut oil and butter-rich meals (*P* < 0.001; Table 4). There was also a significant test fat/oils by time interaction for the postprandial GLP-1 responses (*P* = 0.004; Figure 3B) with the iAUC (but not AUC) found to be significantly lower following the butter containing meals compared to both the coconut oil and vegetable oil-rich meals (*P* = 0.012; Table 4). Similarly, the iAUC for the PYY response was lower after butter than coconut oil-containing meals, although the response following the vegetable oil-rich meals did not differ significantly from either butter or coconut oil (*P* = 0.029; Table 4). No differences were found in postprandial ghrelin or insulin responses or various parameters used to assess perceived satiety responses (hunger, satisfaction, fullness, desire to eat, and prospective consumption) between the test fat/oils (Table 5).

**TABLE 5:** Summary measures for the perceived satiety responses after sequential meals rich in butter, vegetable oils, and coconut oil in healthy males^1^.

	Butter	Vegetable oils	Coconut oil	*P* value^2^
Perceived satiety responses (mm × 480 min)				
Hunger				
AUC	2006 ± 264	2048 ± 301	2026 ± 291	0.773
iAUC^3^	528 ± 197	907 ± 216	633 ± 199	0.579
Satisfaction				
AUC	2753 ± 265	2557 ± 268	2741 ± 254	0.929
iAUC	543 ± 185	893 ± 238	457 ± 186	0.677
Fullness				
AUC	2508 ± 236	2282 ± 235	2458 ± 260	0.932
iAUC	970 ± 243	1188 ± 266	1084 ± 203	0.917
Desire to eat				
AUC	1997 ± 235	2161 ± 309	2124 ± 290	0.813
iAUC	546 ± 142	9604 ± 241	796 ± 196	0.716
Prospective consumption				
AUC	2530 ± 178	2704 ± 243	2531 ± 281	0.840
iAUC	741 ± 220	854 ± 213	935 ± 259	0.604

Abbreviation: iAUC, incremental AUC.

1 Values are untransformed and unadjusted means ± SEMs, *n* = 13 for all variables.

2 Data were analyzed using 1-way analysis of covariance adjusted for baseline (0 min) values. *P* ≤ 0.05 was considered a threshold for statistical significance and adjusted for multiple comparisons using the Bonferroni correction.

3 Indicates data were transformed prior to analysis.

### Postprandial blood pressure, and arterial stiffness

The postprandial time course profiles and summary measures for blood pressure and arterial stiffness did not differ between the test fat/oils (Supplemental Table 1).

## Discussion

Our study has generated novel insights into the effects of plant and animal-based SFA-rich oils on postprandial lipemia, gut hormones, and satiety compared with vegetable oil. Although there were no differences in postprandial TAG (primary outcome measure) between the fats/oils, the postprandial GIP response was shown to be greater after the vegetable oil compared to both butter and coconut oil. Interestingly, the iAUC for PYY and GLP-1 were lower after the butter-rich compared to the coconut oil and vegetable oil (GLP-1 only)-rich meals, but these findings did not translate into differences in perceived satiety from the VAS.

Many of the studies examining the relationship between coconut oil and CVD risk have measured fasting lipids, with a limited number determining the effects on postprandial lipemia. A higher postprandial TAG response has been associated with a greater CVD risk [1,27], and considered to be more discriminatory of risk than fasting TAG. The lack of an effect of the sequential high-fat meals rich in coconut oil, butter, and vegetable oil on the postprandial TAG response is in line with previous findings showing similar lipemic responses after meals rich in coconut oil compared with unsaturated fatty acids [17,29] and animal-derived SFA [16]. However, in contrast, 3 studies have reported postprandial TAG to be lower after coconut oil (30–40 g) compared with other SFA-rich oils (butter, lard, or palm oil) and a palm oil-rich mixture in healthy young adults [12,13,30] whereas a greater response was observed over 180 min after a test meal containing 20 g of coconut than corn oil in adolescents with obesity (11–18 y) [14]. Subgroup analysis according to sex in the Sciarrillo et al. [29] study revealed a greater postprandial TAG response in males than females, which was attributed to the 10 kg/m^2^ difference in BMI between the sexes. Furthermore, the subject groups that have demonstrated lower TAG responses after coconut oil intake were predominantly of Asian origin and consumed coconut oil more regularly in the diet [30]. Adaptive effects of higher habitual intakes may have impacted the rate of postprandial handling of the coconut oil versus animal-derived SFA compared with Caucasian populations. Although differential effects of the plant and animal-derived SFA on postprandial TAG compared with vegetable oil were not found in the current study, it cannot be ruled out that the sex, ethnicity, BMI, or habitual fat intake of the subject group (key determinants of the postprandial lipemic response [10]) may have influenced the findings. Therefore, it would be of interest to compare populations who consume coconut oil habitually, such as populations from South Asia, with those consuming more Western-style diets to determine the effects of coconut oil on fasting and postprandial CVD risk markers.

Meal fat composition has been shown to impact postprandial gastrointestinal hormone responses, but findings have been inconsistent [10]. In our study, vegetable oil-containing meals elicited a higher postprandial GIP response than butter and coconut oil whereas differential effects of the SFA-rich fats/oils were evident for the GLP-1 (vegetable oil and coconut oil > butter) and PYY (coconut oil > butter) responses. These findings align in part with those of Thomsen et al. [31] who reported higher concentrations of GIP and GLP-1 after a MUFA-rich oil [olive oil test meal (80 g)] than coconut oil test meal (100 g) among healthy subjects. In contrast to our study, postprandial PYY concentrations were higher after PUFA-rich vegetable oils (e.g., corn oil and mixture of sunflower and flaxseed oils) than coconut oil in adolescents with obesity [14] and both MUFA-rich (mixed extra virgin olive oil and canola oil) and SFA-rich (mixed butter, palm oil, and coconut oil) meals in females with obesity [32]. Secretion of GLP-1 in the small intestine is dependent on vagal function, with differences in the regulation reported between subjects with and without type 2 diabetes [33]. This might provide a potential explanation for the conflicting outcomes observed in our study conducted in males without obesity and the studies conducted in subjects with obesity. Furthermore, differences in age and sex might also contribute to the disparities in findings.

Gastrointestinal hormones play a key role in modulating satiety response [34]. Ghrelin is an appetite hormone that triggers hunger whereas GLP-1 and GIP are involved in promoting satiety [[35], [36], [37]]. Although ghrelin responses were similar after the test fat/oils, the sequential butter-rich meals were associated with lower postprandial PYY and GLP-1 responses compared with coconut oil, and lower GLP-1 compared with vegetable oil. Despite the differences in these gastrointestinal hormones, satiety ratings were similar between the test fat/oils. A similar finding by Stevenson et al. [32] also suggested that satiety responses (hunger, fullness, and prospective consumption) did not differ between high-fat meals (with 70% of total energy from fat) containing SFA (mixed butter, palm oil, and coconut oil), PUFA (mixed sunflower and flaxseed oil), and MUFA (mixed extra virgin olive oil and canola oil) over 300 min. Similarly, Kinsella et al. [38] and Yao et al. [19] found no differences in postprandial appetite ratings between coconut oil and a vegetable oil, as well as no differences in ad libitum food intake. Although butter contains ∼25% MUFA, differences in satiety following consumption of butter have been shown in other work indicating that butter does not have the same satiating effect as unsaturated fats (sunflower oil and olive oil) [19]. However, this study conducted by 1 of the authors did not include the measurement of gastrointestinal hormones. Research comparing shea oil (saturated fat) to unsaturated fats (canola and safflower) and control (no fat) found that only the unsaturated fats increased fullness and reduced hunger despite no differences in PYY [39]. A possible explanation for the lack of difference in perceived satiety response between the test fat/oils in the present study might be the high-fat content of the sequential meals (80 g in total) consumed during the study visit, which may have suppressed their appetite regardless of the fat type. Measurement of ad libitum food intake was not undertaken in this study, however, based on the similarities in the VAS ratings it is likely that there would be no differences.

Few studies have compared coconut oil with other dietary fats on glucose and insulin responses in which to relate our findings. A systematic review and meta-analysis that included 6 acute studies reported lower postprandial insulin and higher glucose responses with coconut oil [40]. However, most of the studies included in this review compared coconut fat with a control (no fat) or dairy fat, which does not reflect the comparator oils used in the current study. One of the mechanisms by which dietary fatty acids regulate glucose and insulin homeostasis is through their influence on incretin hormones GIP and GLP-1, secreted by the gut in response to nutrient ingestion. Both hormones act directly on pancreatic islets to stimulate insulin secretion in a glucose-dependent manner, thereby supporting postprandial glucose regulation [[41], [42], [43]]. The chain length and composition of dietary fat can modulate the secretion of these hormones, with long-chain fatty acids considered to stimulate greater incretin responses than short- and medium-chain fatty acids [44,45]. This is thought to be attributed to the differences in the rate of absorption of dietary fats in the intestine. Although we did not observe a significant difference in glucose and insulin following the sequential meal containing the different test oils, our findings are in agreement with a previous study which also showed a lack of a relationship between GIP with glucose and insulin responses following test meals rich in SFA (palm olein), MUFA and n-6 PUFA [46]. The authors proposed that the absence of a relationship could be due to a possible role of GIP in other metabolic pathways (such as lipogenesis and lipolysis) other than the insulinotropic effect. We have previously reported greater elevations in postprandial NEFA arising from differences in the metabolism of TAG-rich lipoproteins following meals rich in SFA compared with unsaturated fatty acid-rich meals to be associated with unfavorable effects on glycemia [9]. However, the SFA-rich meal contained palm oil, high in palmitic acid, which has been shown to impair insulin sensitivity in the intestine and liver [44]. The lower palmitic acid content of our SFA-rich butter and coconut oil compared with palm oil may provide an explanation for the effects on GIP but not postprandial glucose and insulin but further studies are needed to confirm these mechanisms.

There were some strengths to the present study. First, only Caucasians males were recruited to eliminate the confounding effects of sex and ethnicity on postprandial lipemia. Second, refined test oils, such as coconut oil, safflower oil, and olive oil, were utilized to eliminate the possible effects of polyphenols within the unrefined oils from impacting glycemia and insulin response. However, the narrow focus on generally healthy Caucasian males may limit its relevance for females and individuals with CVD risk factors, reducing the generalizability of the findings. Additionally, the longer interval between study visits, necessitated by COVID-19 restrictions, could be considered a limitation, though it did not affect fasting data across the 3 visits. Furthermore, a single-blind design was used, which may have introduced bias during data collection and interpretation, as 1 of the researchers (GW) was aware of the type of intervention provided on each study visit. A double-blind design would have minimized such bias, but this was not possible due to the type of dietary fats/oils used, because butter and coconut are solid at room temperature. Although no formal sample size calculation was performed for the secondary outcomes, significant secondary findings should be considered tentative, requiring future validation.

In conclusion, the present study observed that the sequential test meals rich in SFA or vegetable oil did not alter postprandial TAG, NEFA, insulin, and glucose responses or perceived measures of appetite in generally healthy males. However, novel differential effects on gut hormone responses between the animal- or plant-based SFA and vegetable oil were observed, warranting further exploration of the underlying mechanisms.

## Author contributions

The authors’ responsibilities were as follows – JAL, KGJ, GW: designed the human study; GW, DR: conducted the research, encompassing hands-on experimentation and data collection; GW, KGJ: performed sample analysis; GW: analyzed data and performed statistical analysis under the supervision of KGJ and wrote the manuscript, which was reviewed and edited by all co-authors; MEC: provided experimental and statistical advice on gut hormones and satiety responses; JAL, KGJ: provided overall supervision for the research; and all authors: read and approved the final manuscript.

## Data availability

The data that support the findings of this study are available from the corresponding author on reasonable request.

## Funding

The authors reported no funding received for this study.

## Conflict of interest

JAL is Deputy Chair of the UK Scientific Advisory Committee for Nutrition (SACN) and was an expert on SACN’s Saturated Fats Working Group. The views expressed in the manuscript are those of the author and do not necessarily represent those of SACN or the Department of Health and Social Care. The other authors have no conflicts to declare.
