# Supplementary material for: Sequential Meals Containing Animal and Plant-Based Saturated Fats Have Differential Effects on Postprandial Gut Hormones but No Impact on Satiety Compared with Unsaturated Fats in Generally Healthy Males: Findings from the Randomized Controlled Crossover CocoHeart Study
Source: J Nutr. 2025 Jul 1;155(9):3020–9. doi: 10.1016/j.tjnut.2025.06.027 (PMC12799432; doi:10.1016/j.tjnut.2025.06.027)
Supplement: multimedia component 1 [file mmc1.docx]

### **Supplementary table 1**: Postprandial summary measures for blood pressure, hemodynamic, and arterial stiffness responses after sequential meals rich in butter, vegetable oils and coconut oil in healthy men^1^

|  | Butter | Vegetable oils | Coconut oil | *p*-value^2^ |
| --- | --- | --- | --- | --- |
| **Blood pressure**  Systolic blood pressure |  |  |  |  |
| AUC (mmHg x 480 min) | 58268 ± 1107 | 58711 ± 1050 | 58758 ± 1255 | 0.937 |
| iAUC (mmHg x 480 min)* | 77 ± 676 | 1148 ± 982 | -355 ± 882 | 0.663 |
| Diastolic blood pressure |  |  |  |  |
| AUC (mmHg x 480 min) | 39241 ± 971 | 38420 ± 784 | 38998 ± 1071 | 0.735 |
| iAUC (mmHg x 480 min) | -820 ± 573 | -460 ± 775 | -1396 ± 578 | 0.537 |
| Pulse pressure |  |  |  |  |
| AUC (mmHg x 480 min) | 18728 ± 604 | 19725 ± 868 | 19642 ± 588 | 0.567 |
| iAUC (mmHg x 480 min) | 837 ± 697 | 1965 ± 648 | 1009 ± 811 | 0.568 |
| Heart rate |  |  |  |  |
| AUC (beats/min x 480 min) | 28150 ± 1073 | 28894 ± 979 | 28972 ± 1135 | 0.818 |
| iAUC (beats/min x 480 min) | -127 ± 423 | 880 ± 572 | 303 ± 661 | 0.393 |
| Mean arterial pressure |  |  |  |  |
| AUC (mmHg x 480 min) | 47656 ± 1021 | 47708 ± 930 | 48056 ± 1237 | 0.986 |
| iAUC (mmHg x 480 min) | -780 ± 542 | 3131 ± 944 | -1297 ± 695 | 0.529 |
| **Arterial Stiffness** |  |  |  |  |
| Augmentation index |  |  |  |  |
| AUC (% x 480 min) | 4455 ± 1036 | 4954 ± 1042 | 5305 ± 1399 | 0.755 |
| iAUC (% x 480 min) | -2789 ± 2912 | -64 ± 1758 | -368 ± 1365 | 0.475 |
| Pulse wave velocity |  |  |  |  |
| AUC (% x 480 min) | 3771 ± 167 | 3831 ± 187 | 3847 ± 192 | 0.965 |
| iAUC (% x 480 min) | -35 ± 36 | 5 ± 43 | -31 ± 29 | 0.954 |

^1^Values are untransformed and unadjusted means ± SEMs, n=13 for all outcomes presented. Abbreviations: iAUC, incremental AUC; MAP, mean arterial pressure; PWV, pulse wave velocity

^2^Data were analyzed using a linear mixed model to calculate overall treatment effect in postprandial summary measures, with adjustments made for fixed effects of test fat/oils and period. Participant was included as a random effect. *P* ≤ 0.05 was considered a threshold for statistical significance and adjusted for multiple comparisons using the Bonferroni correction..

*Indicates data were transformed prior to analysis
